# Cellular origins of regenerating liver and hepatocellular carcinoma

**DOI:** 10.1016/j.jhepr.2021.100416

**Published:** 2021-12-13

**Authors:** Ágnes Holczbauer, Kirk J. Wangensteen, Soona Shin

**Affiliations:** 1 Division of Gastroenterology and Hepatology, Perelman School of Medicine, University of Pennsylvania, Philadelphia, Pennsylvania, USA; 2 Liver Tumor Program, Division of Pediatric General and Thoracic Surgery, Cincinnati Children’s Hospital Medical Center, Cincinnati, Ohio, USA; 3 Department of Surgery, University of Cincinnati College of Medicine, Cincinnati, Ohio, USA

**Keywords:** Hepatocellular carcinoma, lineage tracing, carcinogenesis, hepatocyte, progenitor cell, BEC, biliary epithelial cell, CCl_4_, carbon tetrachloride, CDE, choline-deficient, ethionine-supplemented, DDC, 3,5-diethoxicarbonyl-1,4-dihydrocollidine, DEN, diethylnitrosamine, EpCAM, epithelial cell adhesion molecule, Foxl1, forkhead box L1, HB, hepatoblastoma, HCA, hepatocellular adenoma, HCC, hepatocellular carcinoma, HDTVI, hydrodynamic tail vein injection, HNF, hepatocyte nuclear factor, HPC, hepatic progenitor cell, HybHP, hybrid hepatocyte, Krt19, cytokeratin 19, OPN, osteopontin, PH, partial hepatectomy, Sox9, SRY-box 9, TAA, thioacetamide, ICC, intrahepatic cholangiocarcinoma, Fah, fumarylacetoacetate hydrolase, NASH, non-alcoholic steatohepatitis, HBV, hepatitis B virus, HCV, hepatitis C virus, FLC, fibrolamellar carcinoma

## Abstract

Hepatocellular carcinoma (HCC) is the predominant primary cancer arising from the liver and is one of the major causes of cancer-related mortality worldwide. The cellular origin of HCC has been a topic of great interest due to conflicting findings regarding whether it originates in hepatocytes, biliary cells, or facultative stem cells. These cell types all undergo changes during liver injury, and there is controversy about their contribution to regenerative responses in the liver. Most HCCs emerge in the setting of chronic liver injury from viral hepatitis, fatty liver disease, alcohol, and environmental exposures. The injuries are marked by liver parenchymal changes such as hepatocyte regenerative nodules, biliary duct cellular changes, expansion of myofibroblasts that cause fibrosis and cirrhosis, and inflammatory cell infiltration, all of which may contribute to carcinogenesis. Addressing the cellular origin of HCC is the key to identifying the earliest events that trigger it. Herein, we review data on the cells of origin in regenerating liver and HCC and the implications of these findings for prevention and treatment. We also review the origins of childhood liver cancer and other rare cancers of the liver.

**Key points**

• Mature hepatocytes can generally divide to regenerate liver mass after injuries, but in the setting of severe hepatocyte injuries, hepatic progenitor cells (HPCs) may step in to generate hepatocytes.
• Both HPCs and mature hepatocytes have been shown to have the capacity to form HCC.
• Lineage tracing experiments performed in mice have indicated that in most conditions HCC arises from mature hepatocytes.
• Additional research is needed to determine whether any specific subset of hepatocytes tends to contribute more to tumorigenesis than others, and whether any cell type can be targeted to prevent HCC.

## Introduction

Hepatocellular carcinoma (HCC) is the predominant primary cancer of the liver and causes 790,000 deaths annually worldwide.^1^^,^^2^ Major risk factors include chronic hepatitis B virus (HBV) and hepatitis C virus (HCV) infection, heavy alcohol intake, and the metabolic syndrome, a collection of conditions that includes insulin resistance, obesity, hyperlipidaemia, and hypertension, which is associated with non-alcoholic steatohepatitis (NASH).^3^^,^^4^ In more than 90% of cases, HCC occurs on a background of cirrhosis, whereas in a minority of cases, particularly with HBV infection and increasingly with NASH, it develops in livers with minimal or no fibrosis.^5^^,^^6^ Patients with cirrhosis have annual incidence rates of HCC of 2-4%.^7^ Other risk factors include toxins, autoimmune hepatitis, cholestatic liver diseases, hereditary haemochromatosis, and α1-antitrypsin deficiency.^8^ A family history of HCC is an independent risk factor for HCC, and cancer-associated genetic variants can be detected in the germline of patients with HCC, but the full spectrum of underlying genetic risk factors is not yet known.^6^^,^^9^

Hepatocytes are the main parenchymal cells of the liver, representing 80% of the total liver volume. Although hepatocytes in the adult liver rarely divide under normal conditions, they have tremendous regenerative capacity upon liver injury. Hepatocytes are heterogeneous, performing distinct metabolic functions depending on their location within the liver lobule – periportal (zone 1), midlobular (zone 2), or pericentral (zone 3) – a phenomenon called zonation.[10], [11], [12], [13] Approximately 50% of human and 90% of mouse hepatocytes are polyploid, with polyploid cells spread across all zones.^13^^,^^14^

Many cell types other than hepatocytes exist in the liver. The other parenchymal cells of the liver, the biliary epithelial cells (BECs) or cholangiocytes, form the tree-like three-dimensional structure of the intrahepatic biliary system. Non-parenchymal cells include liver sinusoidal endothelial cells, stellate cells, portal fibroblasts, immune cells, and resident macrophages, also known as Kupffer cells.^15^

In liver injury, there is an expansion of an oval-shaped population of BECs in association with the bile ducts and their terminal branches, the canals of Hering, which is known as the ductular reaction.[16], [17], [18] Ductular reactions are observed in acute and chronic hepatocellular and cholestatic liver injuries.^16^^,^[19], [20], [21], [22] The cells of the ductular reaction express biliary proteins, and, in certain settings, have been shown to have a bipotential capacity to supply both hepatocytes and BECs, as a facultative stem cell compartment.[23], [24], [25], [26], [27], [28], [29] The term hepatic progenitor cell (HPC) is used to describe these oval-shaped cells in ductular reactions. The hallmarks of HPCs include high turnover, ability to self-renew, and capability of bidirectional differentiation into hepatocytes and BECs.^28^^,^^29^ However, unlike organs such as the intestine and skin that rely on tissue-resident stem cells for homeostasis, the predominant evidence supports pre-existing, fully differentiated hepatocytes as the main source of cells for homeostasis, regeneration after injury, and HCC.

There has been continuous debate about whether all or only a specific subset of HPCs or hepatocytes are responsible for liver maintenance, regeneration, and HCC. The development of effective off/on switches for reporter genes has enabled *in vivo* tracking of specific cell types to trace their contribution to tissues. Inducible Cre systems have allowed researchers to mark HPCs or hepatocytes with a reporter, such as GFP, followed by induction of liver injury or carcinogenesis. Cell lineage tracing also provided fundamental insights into the molecular mechanisms governing cell fate decisions.

Herein, we review the evidence for HPCs and hepatocytes as cells of origin for the regenerating liver and for the development of HCC. We discuss areas of controversy and areas requiring further investigation, and we propose that there may be significant plasticity in cellular origins that depends on the context and the oncogenic drivers of HCC.

## Origin of regenerating cells that respond to liver injury

The liver has a unique capacity to regenerate after acute or chronic liver injury to ensure sufficient liver mass for homeostasis. Highly redundant autocrine, paracrine, and endocrine signals coordinate liver regeneration. Regenerative responses are proportional to the severity of injury up to a threshold, beyond which injury progresses to liver failure and death. Partial hepatectomy (PH), the surgical removal of a portion of the liver, is the most widely used rodent model of liver regeneration to date. In rodents, complete restoration of liver mass occurs within 3 weeks after a two-third PH. Unlike PH, the ischemia/reperfusion injury model relies on the reduction of functional liver mass via anoxia, while the structure of the liver is relatively unaffected. Chemical injury models utilise a variety of hepatotoxic chemicals to induce cellular death and compensatory liver regeneration, including thioacetamide (TAA), chloroform, carbon tetrachloride (CCl_4_), choline-deficient, ethionine-supplemented (CDE) diet, bromobenzene, 3,5-diethoxicarbonyl-1,4-dihydrocollidine (DDC), acetaminophen, trichloroethylene, allyl alcohol, and galactosamine.[30], [31], [32], [33], [34], [35], [36], [37]

### Biliary epithelial cells and hepatic progenitor cells as cellular origins of regenerating liver

While HPCs are detected in nearly all liver diseases,^16^^,^^21^ subsequent differentiation of HPCs into hepatocytes or BECs that contribute to the restoration of liver mass and function, and vice versa (*i.e.* reprogramming of hepatocytes into BECs), is only observed in specific contexts.^38^ Tracing of BECs and HPCs has most often been performed using the biliary/progenitor markers SRY (sex-determining region Y)-box 9 (Sox9), cytokeratin 19 (Krt19), osteopontin (OPN), hepatocyte nuclear factor (HNF)1β, and forkhead box L1 (Foxl1).[39], [40], [41], [42], [43], [44], [45], [46], [47], [48], [49]

#### Sox9+ cells

Sox9-positive liver cells have been reported to replenish hepatocytes during homeostasis and injury, but conflicting evidence has raised doubts about whether these are BECs, HPCs, or periportal hepatocytes.

Using lineage tracing of Sox9-expressing cells during foetal development and in the postnatal period, a report suggested that a number of periportal hepatocytes were derived from Sox9-labelled cells, however, overall maintenance of the hepatocytes occurred without continuous generation from Sox9-labelled cells.^44^ In contrast, another report proposed that Sox9+ BECs functioned as progenitors that continuously replenished hepatocytes during physiological homeostasis.^49^ Additional work to trace Sox9+ HPCs using multicoloured fluorescent Confetti reporter mice showed that Sox9+ cells clonally expanded but rarely produced hepatocytes following chronic liver injury induced by CDE, DDC, or CCl_4_.^50^ A separate group reported that Sox9^low+^ hepatocytes found in the periportal zone significantly contributed to the restoration of liver parenchyma after chronic liver injury induced by CCl_4_.^51^ In line with these findings, co-labelled periportal cells expressing the hepatocyte marker HNF4α plus Sox9 acted as bipotent progenitor cells after liver injury, giving rise to both hepatocytes and BECs.^52^

Thus, Sox9^+^ cells may contribute to liver repopulation, but Sox9 is not a very specific lineage label as it captures periportal hepatocytes, BECs, and HPCs.^53^ There have been discrepant results with Sox9 lineage labelling that may be related to the dose of tamoxifen,^44^ leaky nuclear translocation of CreERT2,^51^ the type and severity of injury,^44^^,^^50^^,^^51^ and differential designs of genetic models – transgenic^44^^,^^50^^,^^51^^,^^54^ *vs*. knock-in.^49^ Overall, lineage labelling with Sox9 has demonstrated plasticity between BECs, HPCs, and hepatocytes.

#### OPN+ cells

Lineage tracing of OPN-expressing BECs and HPCs found that there were no label-positive hepatocytes during 6 months of liver homeostasis, nor during liver regeneration following PH or acute toxic injury induced by CCl_4_.^42^ However, OPN^+^ HPCs and/or BECs generated 2.45% of hepatocytes by the end of a 2-week recovery after CDE diet-induced chronic hepatocellular injury. Similarly, OPN labelling of biliary cells followed by long-term (>24 weeks) CCl_4_ treatment resulted in 12% of hepatocytes being label-positive.^46^

#### HNF1β+ cells

When HNF1β+ biliary cells were lineage labelled, they did not result in any label-positive hepatocytes during liver homeostasis or following PH or acute acetaminophen and CCl_4_ injury. Similarly, no HNF1β+ cell-derived hepatocytes were observed after DDC- or CCl_4_-induced chronic liver injury. On the other hand, 1.86% of total hepatocytes were derived from HNF1β+ cells following a CDE diet.^43^ Thus, HNF1β+ biliary cells contributed to liver regeneration in a liver injury model-dependent manner.

#### Foxl1+ cells

An author of this review previously found that Foxl1 is a marker for HPCs in murine postnatal livers, and their descendants form hepatocytes after a recovery period following CDE diet feeding.^28^^,^^48^ The severity of liver injury beyond a certain threshold was critical for HPC-to-hepatocyte transdifferentiation. In severely injured mice, up to 29% of hepatocytes were Foxl1+ lineage labelled, indicating an HPC origin.

#### Krt19+ cells

In mice treated with TAA or DDC for 24 weeks, 10% and 9.1% of hepatocytes were Krt19-positive, respectively, indicating BECs as the cellular origin.^39^ Interestingly, BEC-to-hepatocyte conversion occurred via HNF4α+/Krt19+ biphenotypic cells, which did not express HPC markers, implicating a conversion without an intermediate progenitor state.

#### Contribution of BECs and HPCs to liver regeneration in combined injury models

Several groups used the strategy of overexpressing or deleting specific genes in hepatocytes to inhibit their proliferation in the context of chemical liver injury. In these combined injury models, the contribution of BECs/HPCs to hepatocyte number greatly exceeded the levels seen in models of chemical injury alone. For example, loss of β1-integrin or overexpression of p21 in murine hepatocytes in combination with liver damage induced by DDC, TAA, CDE, or methionine- and choline-deficient diet triggered ductular reactions followed by the appearance of BEC-derived hepatocytes.^41^ Similarly, hepatocyte-specific deletion of Mdm2 or β-catenin provoked the differentiation of BEC-derived HPCs or BECs into hepatocytes upon severe liver injury.^45^^,^^55^

#### Role of the microenvironment in cell fate decisions of hepatic progenitor cells

Local signalling plays an important role in cell fate decisions of HPCs. The Wnt/β-catenin pathway is associated with HPC activation and differentiation toward hepatocytes, while Notch signalling in HPCs is linked to differentiation toward BECs.^26^^,^[56], [57], [58] Clearing of hepatocyte debris induced Wnt3a expression in macrophages in CDE- or DDC-treated mice, which led to the activation of the canonical Wnt pathway in HPCs, promoting their differentiation into hepatocytes. On the other hand, expression of the Notch ligand Jagged1 by myofibroblasts activated Notch signalling in HPCs and promoted their differentiation into BECs during biliary regeneration.^23^

Changes to the extracellular matrix during chronic liver injury are crucial for HPC activation and differentiation. Depletion of laminin, a key extracellular matrix component of the HPC niche, increased the number of HPC-derived hepatocytes in CDE-treated mice.^42^ Disruption of hepatocyte growth factor/MET signalling in the setting of DDC altered the composition of the HPC microenvironment, decreased HPC numbers, and led to liver failure.^59^ In contrast, liver-specific conditional knockout of the epidermal growth factor receptor led to increased expansion of HPCs in response to DDC, and the HPCs tended toward hepatocyte rather than BEC differentiation, suggesting that epidermal growth factor receptor signalling directs BEC differentiation.^60^

A crucial component of the microenvironment is fibrosis and eventually cirrhosis that develops with chronic liver injuries and may affect cellular plasticity. Recent data suggest that expansion of HPCs in cirrhotic livers gives rise to regenerative nodules.^61^ Furthermore, mitochondrial DNA mutation analysis of human liver tissues revealed that HPCs and regenerative nodules shared identical mutations, indicating common origins.^62^^,^^63^

### Hepatocyte cellular origins of regenerating liver

Multiple lines of evidence indicate that the predominant cells of origin of new hepatocytes in liver homeostasis and regeneration are pre-existing hepatocytes. Lineage tracing of hepatocytes following PH and various HPC-inducing toxic liver injuries in AAV8-*TBG*-*Cre*-injected *R26*^*YFP*^ mice showed that the percentage of labelled hepatocytes remained unchanged at more than 99% following liver injuries, implicating liver repopulation by pre-existing hepatocytes.^64^ Furthermore, Krt19+ biliary/progenitor cells were genetically labelled using *Krt19-CreER;R26*^*YFP*^ mice. Under HPC-inducing injury or homeostatic conditions, all YFP+ cells coexpressed Krt19 but not the hepatocyte marker HNF4α, indicating that YFP+ biliary/progenitor cells did not contribute to hepatocytes.^64^ Similar results were obtained in a separate hepatocyte lineage tracing study using *Alb-DreER;R26-RSR-tdTomato* mice, in which more than 99.5% of hepatocytes were genetically labelled following tamoxifen-induced Dre-rox recombination. After PH and chemically induced chronic liver injury, almost all hepatocytes were tdTomato+ in regenerated and control livers, demonstrating that new hepatocytes originated from pre-existing hepatocytes.^65^

#### Significance of hepatocyte ploidy in liver regeneration

Recent studies suggest differential roles for diploid and polyploid hepatocytes in homeostasis and regeneration. Importantly, hepatocyte polyploidisation is a dynamic process, as diploid hepatocytes may become polyploid and polyploid hepatocytes may become diploid during cell division, a phenomenon termed the ploidy conveyor.[66], [67], [68], [69], [70] Mice lacking the transcription factors E2f7 and E2f8 in the liver have mostly diploid hepatocytes, and therefore avoid the ploidy conveyor phenomenon.^67^ E2f7/E2f8-deficient hepatocytes (mostly diploid) proliferate faster and massively outcompete control hepatocytes (mostly polyploid) in competitive repopulation studies. During PH, although both the diploid and polyploid hepatocyte population contributed to liver regeneration, diploid hepatocytes entered the cell cycle earlier and progressed through faster compared to polyploid hepatocytes. Similar findings were reported in a study using heterozygous *Rosa26-Rainbow* reporter mice and AAV8-*TBG*-*Cre* to randomly label hepatocytes across the liver lobule. This study demonstrated a broad distribution of hepatocytes that proliferate and contribute to normal liver tissue maintenance and regeneration in acute or chronic liver injury, challenging the concept of a specialised liver stem cell compartment. Notably, diploid hepatocytes replicated more efficiently than polyploid hepatocytes after chronic injury induced by CCl_4_.^68^

Taking an elegant multicolour reporter allele system, the heterozygous *Ubc-CreERT2;Rosa-Confetti*^*+/-*^ mice, polyploid hepatocytes can be labelled as multicoloured, whereas diploid cells express only a single reporter gene after Cre recombination. Polyploid hepatocytes showed extensive repopulation capability, ploidy reduction, and repolyploidisation when transplanted into fumarylacetoacetate hydrolase (*Fah*)^-/-^ recipient livers. Furthermore, proliferation of polyploid hepatocytes and ploidy reduction also occurred after liver injury induced by CCl_4_, DDC, TAA, and Fah-deficiency.^69^

Thus, although polyploid hepatocytes may have a slightly reduced ability to regenerate, the ploidy state is dynamic, and polyploid hepatocytes can divide to form diploid cells, and then later form new polyploid cells.

#### Hepatocyte zonation and contribution to regenerating liver

**Fig. 1:**
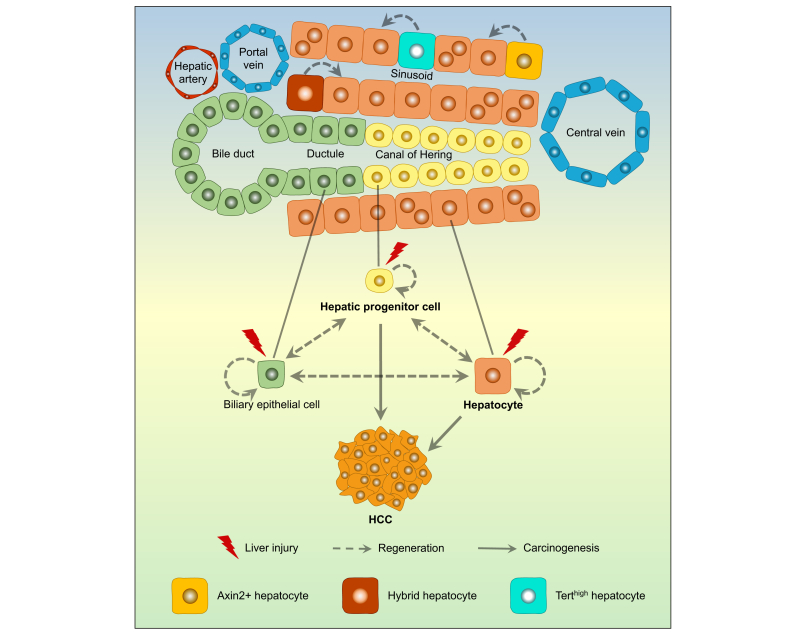
Cellular origins of liver regeneration and HCC. In the quiescent state, liver cells divide infrequently. In the setting of liver injuries such as infection with HBV or HCV, or exposure to liver toxins, hepatocytes divide to restore lost parenchyma, and the population of hepatic progenitor cells, which are associated with the canals of Hering and bile ductules, expands. Evidence from lineage tracing and forced expression of oncogenes in various cell types suggests that hepatic progenitor cells and hepatocytes both have the capacity to generate HCC. The relative contribution of hepatic progenitor cells or hepatocytes to HCC may depend on the type of injury, the specific genetic alterations, and the surrounding microenvironment. HCC, hepatocellular carcinoma.

Besides heterogeneous ploidy states, the different zones of the liver lobule have recently been found to make varying contributions to liver homeostasis and regeneration (see Fig. 1).^51^^,^[71], [72], [73], [74], [75], [76], [77]

As mentioned earlier, periportal hepatocytes (zone 1) of uninjured liver labelled as Sox9^low+^ cells express several biliary/progenitor cell markers.^51^ These cells have been named hybrid hepatocytes (HybHPs). After chronic liver injury using CCl_4_, label-positive HybHPs contributed substantially to the restoration of liver parenchyma.

Pericentral (zone 3) hepatocytes are marked by a characteristic activation of the Wnt-β-catenin pathway, where Wnt signals are provided by adjacent central vein endothelial cells.^71^ A Wnt-responsive, diploid Axin2+ liver cell population located around the central vein, which the authors referred to as hepatocyte stem cells, was found to contribute substantially to normal hepatocyte maintenance.^71^ However, this was challenged in a study that used Axin2 lineage tracing in BAC-transgenic mice to avoid potential *Axin2* haploinsufficiency.^76^ In this study, Axin2+ pericentral hepatocytes did not possess superior proliferative ability, and proliferation of hepatocytes throughout the liver could account for homeostasis and restoration of liver mass after PH. Similarly, rare hepatocytes with high telomerase expression distributed throughout the liver lobule were found to repopulate all zones during homeostasis and regenerate the liver in response to injuries.^74^

A comprehensive recent study used 14 fate mapping *CreER*-based mouse strains to systematically compare distinct subpopulations of hepatocytes during liver homeostasis and injury. This study found significant differences in the repopulation capacity of hepatocytes from different zones. The proportion of periportal (zone 1) hepatocytes declined over 6- and 12-month periods, indicating that the contribution of zone 1 cells to liver homeostasis is negligible. Similarly, pericentral (zone 3) hepatocytes showed no significant contribution. Most importantly, zone 2 hepatocytes preferentially repopulated the liver lobule during normal liver maintenance and when either zone 1 or 3 was damaged by hepatotoxins.^77^

The controversies among studies indicate that an approach based on unbiased labelling is required.^68^ A proliferation tracer (ProTracer) model, which allows an unbiased assessment of proliferative events over time, demonstrated that the hepatocyte proliferation rate was the highest in zone 2 during normal homeostasis, and hepatocytes next to injury sites contributed most to liver regeneration.^78^

In conclusion, the evidence indicates that hepatocytes are the predominant cells of origin of newly formed hepatocytes in normal liver tissue maintenance and regeneration following low-level injury, with little replenishment of hepatocytes from BECs and HPCs. Recent studies put the existence of a specific hepatocyte population acting as a stem cell compartment into question. Rather, all hepatocytes have the potential to step up and repair liver injuries. With certain types of chronic hepatocyte injury, however, distinct populations of BECs and HPCs have some capacity to generate hepatocytes, perhaps as a failsafe in the event of devastating liver injuries.

## Cellular origin of HCC

The cellular origin of HCC has been a topic of debate and research for decades. HCCs are usually found in cirrhotic livers with abundant ductular reactions, and they often express both hepatocyte and biliary markers.[79], [80], [81] The diseased microenvironment of chronic inflammation, continuous hepatocyte destruction, and liver regeneration provides fertile soil for HCC initiation and progression. Mutations and chromosomal aberrations are increased in hepatocytes of patients with cirrhosis, and mutational signatures and epigenetic changes overlap between cirrhotic tissue and HCC.^63^^,^^82^ This is a dynamic state that occurs over many years, and rodents may not adequately recapitulate cirrhosis that develops in humans. As a carcinoma, HCC has an epithelial origin, but it remains unclear whether HCCs originate from damaged hepatocytes, regenerating healthy hepatocytes, or activated HPCs (see Fig. 1).

Rodent models have helped to characterise the cellular origins of HCC. Dating back as far as the 1950s, experiments performed in rats treated with liver carcinogens have identified a process of carcinogenesis that is similar but accelerated in comparison to human carcinogenesis, with HCC developing in association with ductular reaction and fibrosis. These early studies suggested HPCs as the potential cell of origin for HCC.[83], [84], [85]

However, more recent cell lineage tracing experiments implicate hepatocytes as the predominant cell of origin. Like with regenerating liver cells, inducible reporter systems have enabled scientists to trace the cell of origin of HCC by lineage labelling hepatocytes or HPCs with a reporter, followed by induction of hepatocarcinogenesis and examination of resultant tumours for reporter gene expression.

### HPCs as the cell of origin of HCC

It was long assumed that HPCs in the stem/progenitor compartment can contribute to HCC based on correlative observations. First, HCC often exhibits markers and gene expression signatures of stem/progenitor cells, and expression of progenitor markers in HCC is associated with a poor prognosis.[86], [87], [88], [89] However, expression of stem/progenitor cell markers may reflect dedifferentiation of cells derived from mature hepatocytes or phenotypic plasticity of cancer cells. Second, accumulation of HPCs is detected in several liver diseases associated with an increased risk of cancer as well as in livers with HCC both in human and animal models.^16^^,^^19^^,^^29^ Lastly, cancer stem cells in HCC, a subpopulation of cells within a given tumour with capacity for self-renewal and tumorigenesis, share progenitor cell molecular profiles with HPCs.^61^^,^^90^

A study performed in mice by one of the review authors found that hepatoblasts, HPCs, and hepatocytes all have the capacity to form HCC. This study isolated these various primary cell types and transformed them by introducing oncogenic H-Ras and SV40LT, then transplanted them into immunodeficient mice.^90^ Tumours displayed various histological features of HCC, intrahepatic cholangiocarcinoma (ICC), and other tumour phenotypes irrespective of the origin of the transplanted cell types. The tumours also all expressed markers of progenitors/biliary cells including Krt19, epithelial cell adhesion molecule (EpCAM), and A6. This work indicated that these cell types all have the potential to contribute to HCC. It also showed that marker expression cannot be used to extrapolate the cell type of origin of HCCs, as, for instance, hepatocytes could form tumours with biliary cell marker expression.

In support of HPCs with a capacity to develop HCC, lineage tracing of a population of liver cells that expresses EpCAM upon liver injury, which the authors termed proliferating ductal cells, could give rise to HCC.^91^ Because the *Epcam*^*CreERT2*^ transgene did not label quiescent cells in chow-fed animals, lineage labelling with tamoxifen was performed only after initiation of injury with DDC treatment. Using activation-induced cytidine deaminase (AID) conditional transgenic (cTg) mice to induce genetic alterations, the *Epcam*^*CreERT2*^*;AID* cTg mice developed label-positive HCCs. About half of the tumours had sparse ductule-like cholangiolocellular features, suggesting they formed combined hepatocellular-cholangiocarcinoma (cHCC-ICC). In comparison, HCCs that emerged from inducing mutations in hepatocytes using *Alb*^*Cre*^*;AID* cTg mice were indistinguishable from the HPC-derived tumours except that they had no ductule-like structures. In short, HPCs have the capacity to form HCC after the acquisition of genetic alterations.

### Hepatocytes as the cell of origin of HCC

The long life span and remarkable regenerative potential of mature hepatocytes strongly support their susceptibility to malignant transformation under selective pressure induced by chronic inflammatory cell death.^92^ This concept is supported by various mouse models of hepatocarcinogenesis, especially by those established using hydrodynamic tail vein injection (HDTVI), which predominantly induces genetic alterations in mature hepatocytes.^93^^,^^94^ For example, coexpression of MET or N-Ras^G12V^ and activated β-catenin using HDTVI can induce HCC formation with 4- or 13-week latency, respectively.^95^^,^^96^ Several models combine HDTVI with the *Fah*-null mouse model of liver repopulation. In these models, HCC development is driven by selective repopulation of Fah-positive hepatocytes expressing the transfected genes and by the cytotoxic microenvironment of *Fah* mutant livers.[97], [98], [99] Sequential phenotypic changes in diseased liver, such as the emergence of dysplastic foci, nodules, and HCC further support oncogenic transformation of mature hepatocytes.^100^

In a report examining the cell of origin of HCC, lineage tracing of hepatocytes was performed using AAV8-*TBG*-*Cre* viral transduction, followed by induction of HCC by the mutagen diethylnitrosamine (DEN) combined separately with several hepatotoxins: CCl_4_, DDC, and CDE diet.^101^ The resultant tumours were derived solely from hepatocytes. In contrast, lineage tracing of BECs/HPCs using the *Opn-CreERT2* allele showed no contribution to HCC in these conditions.^101^ One of the downsides of toxin-based model systems is that hepatocytes metabolise the toxins, which may affect the results. However, the main risk factors for HCC in humans also derive from hepatotoxic injuries provoked by HBV and HCV, which have a tropism for hepatocytes.^6^ As an alternative to toxin-based hepatocarcinogenesis, genetic models of liver injury, namely the *Mdr2*^KO^ and *Pten*^*fl/fl*^ models, again showed hepatocytes as the predominant source of HCC.^101^ Another group used the biliary marker HNF1β to lineage label BECs prior to the induction of HCC.^102^ Using both the *Mdr2*^KO^ model and the DEN-induced HCC model, hepatocytes were the cell of origin of HCC, as no HNF1β-labelled biliary cells gave rise to tumour cells.

Authors from this review used *Foxl1-Cre* to determine whether a subtype of HPCs can become tumours.^103^ Hepatocarcinogenesis was induced using 2 models that combined DEN with a hepatotoxin, either CCl_4_ or 3,3’,5,5’-tetrachloro-1,4-bis(pyridyloxy)benzene. Notably, the HPC marker Foxl1 is only ever expressed in the liver upon injury, therefore, *Foxl1-Cre* can be used to induce marker gene expression in Foxl1-positive HPCs, which avoids any confounding effects on hepatocarcinogenesis from adding tamoxifen.^104^^,^^105^ None of the tumours that formed in *Foxl1-Cre;Rosa*^*YFP*^ mice treated with hepatotoxins were YFP-positive, indicating that tumours were not derived from the Foxl1-expressing HPCs. Separately, an AAV8-*TBG*-*Cre*-mediated system was used to label hepatocytes, and the HCCs and hepatocellular adenomas (HCAs) that formed were all marker-positive, indicating a hepatocyte origin for HCC in these hepatocarcinogenesis models.

The relative contribution of hepatocytes *vs.* HPCs to liver tumour formation may depend on the injury model.^61^^,^^91^ In the *hURI-tetOFF*^*hep*^ model, hepatocyte-specific expression of hURI (human unconventional prefoldin RPB5 interactor) depleted the energy cofactor NAD+, leading to DNA damage and the development of liver tumours.^61^^,^^106^ This model mimics multistep human hepatocarcinogenesis with the development of focal nodular hyperplasia, regenerative nodules, NASH, HCAs, and HCCs. The serum albumin *SA*^*CreERT2*^*;R26-stop-EYFP* reporter system was used to trace hepatocytes, while the *Sox9*^*IRES-CreERT2*^ line was used to trace ductal cells. Interestingly, hepatocytes were the major cell of origin for HCC and also gave rise to HCA in this model, but Sox9+ ductal cells could also be transformed to various types of malignant and non-malignant lesions including HCC, HCA, and regenerative nodules. This study also demonstrated that, as shown by other groups, hepatocytes were the only cell of origin of HCC in the *Mdr2*^KO^ and DEN/CCl_4_ models. This indicates that the conversion of HPCs into tumour cells may depend on the type of liver damage and model of carcinogenesis.

#### Subpopulations of hepatocytes as the cellular origin of HCC

Subpopulations of hepatocytes have also been examined for their contribution to HCC development, including ploidy state and zonality.

In both humans and rodents, HCCs tend to be diploid rather than polyploid, implicating the polyploid state as possibly tumour protective.^107^^,^^108^ In support of this hypothesis, diploid hepatocytes were found to be susceptible to tumour suppressor loss of heterozygosity, while also being as susceptible to MYC oncogene activation as polyploid hepatocytes, in mouse models of altered hepatocyte ploidy status.^109^ Furthermore, mouse strains with a higher percentage of polyploid hepatocytes developed significantly fewer HCCs following chronic liver injury induced by DEN or CCl_4_ compared to control mice.^70^^,^^110^

In contrast to the studies showing that increased ploidy may be tumour-protective, liver injury with DEN increased the polyploidisation of hepatocytes in the pericentral zone and led to dysplastic foci containing cells undergoing aberrant reduction of ploidy level to promote HCC, suggesting that polyploidisation can be maladaptive.^111^ This notion is compatible with a study demonstrating dynamic ploidy gain and loss in hepatocytes in the process of carcinogenesis, using lineage tracing of polyploid hepatocytes to prove that they have a capacity to contribute to HCC.^112^ Finally, close examination of human HCCs indicated expansion of nuclear ploidy level during tumorigenesis, especially in tumours with *TP53* mutations, which correlated with worse prognosis.^113^

Regarding zonality, recent data support pericentral hepatocytes as the origin for a disproportionate amount of HCCs. Lineage tracing of Lgr5^+^ pericentral hepatocytes, which constitute about 2% of total hepatocytes, demonstrated that these cells give rise to 40% of tumours in a DEN-induced HCC model.^72^ The metabolism of DEN by pericentral hepatocytes might have led to more injury in this zone. However, in support of a pericentral predominance in generating HCCs, periportal HybHPs did not give rise to cancer in various toxin-induced and genetic models of HCC.^51^ This included a model of NASH that affects all zones, suggesting that HypHP cells are incapable of tumorigenesis.^51^ Additional studies to lineage label all 3 zones of hepatocytes are needed to compare their relative contribution to HCC.

#### Role of microenvironment in lineage commitment of transformed hepatocytes

The type of liver injury and consequently the hepatic microenvironment may also play a critical role in the lineage commitment of transformed hepatocytes. A report compared 2 different plasmid delivery systems expressing MYC and N-Ras^G12V^ or MYC and AKT1 in the hepatocytes of *p19*^*Arf*−/−^ mice. Interestingly, plasmid delivery by HDTVI resulted in the development of HCC, whereas plasmid delivery by *in vivo* electroporation induced ICC or cHCC-ICC. Both methods induced tissue damage and an associated inflammatory response with similar infiltrates. However, HDTVI predominantly caused hepatocyte apoptosis, while electroporation induced necroptotic cell death with a specific cytokine microenvironment. Notably, pharmacological or genetic suppression of necroptosis reduced the induction of most electroporation-specific cytokines and switched ICC to HCC development, confirming the decisive role of the necroptotic microenvironment in liver cancer lineage commitment.^114^

### Foetal progenitor cells as the cell of origin of HCC

As mentioned above, hepatoblasts isolated from E16.5 foetal liver and expressing H-Ras and SV40LT can give rise to HCC when injected subcutaneously or orthotopically into immunodeficient mice.^90^ This observation has been further corroborated by work showing that hepatoblasts isolated from *p53* knockout mice at E13.5 and injected into pre-conditioned wild-type mice led to intrahepatic tumours with varied appearances resembling characteristics of cHCC-ICC with stem cell features as well as extrahepatic metastases.^115^ Similarly, when β-catenin was activated in foetal progenitor cells using *Cited1-CreER™* transgenic mice, both HCC and hepatoblastoma (HB) developed in mice.^116^ These studies clearly demonstrate that foetal progenitor cells can give rise to HCC when oncogenes are expressed in them. However, hepatoblasts are seen only in early development and the vast majority of HCCs are seen in adults, so the relevance of foetal progenitor cells to human HCCs remains in doubt.

## Cell of origin for childhood liver cancer

Hepatoblastoma is the most common primary liver cancer in children, usually occurring before age 3.^117^^,^^118^ While several conditions including very low birth weight, Beckwith-Wiedemann syndrome, and familial adenomatous polyposis are associated with an increased risk of HB, the exact aetiology remains unknown in most cases.^119^ Interestingly, HB has a low mutational load, with only 2.9 mutations per tumour. Mutations resulting in the constitutive activation of β-catenin were observed in 60% to 70% of all cases.[119], [120], [121]

To date, only a few relevant *in vivo* models have been generated for HB. Among several histologic subtypes of HBs, these models only simulate the epithelial subtypes.^116^^,^[122], [123], [124], [125] In a mouse model generated by overexpression of constitutively active β-catenin and YAP in adult hepatocytes using HDTVI, rapid development of liver tumours resembling human HB was observed.^122^ Overexpression of MYC or the RNA-binding protein LIN28B in mice resulted in the development of mixed embryonal/foetal or foetal/cholangioblastic HBs, respectively.^123^^,^^124^ In another model, MYC and a dominant mutant allele of β-catenin were coexpressed in immature mouse liver cells. Neonatal mice preferentially developed HBs over HCCs, all of which aligned histologically and molecularly with human HBs.^125^ Similarly, foetal progenitor cells with an activating mutation of β-catenin can give rise to HB as described above.^116^ Collectively, these studies indicate that HB can be derived both from foetal and adult liver cells, and that this may depend on activation of β-catenin, as occurs in humans with familial adenomatous polyposis.

Fibrolamellar carcinoma (FLC) is a rare HCC variant seen in adolescents and young adults without the chronic injuries that usually precede HCC. A DNAJB1-PRKACA fusion kinase resulting from a somatic deletion of ∼400 kilobases on chromosome 19 has been detected in most cases.^126^^,^^127^ Expression of this fusion kinase in adult hepatocytes was sufficient to induce tumours resembling human FLC in mouse livers.^128^^,^^129^ In contrast, the molecular profile of patient-derived xenografts has implicated biliary tree stem cells located in peribiliary glands or hepatic mesothelial progenitors as potential cells of origin for FLC.^130^^,^^131^ As shown with HCC, a variety of hepatic cell types may have the capacity to establish FLC once the oncogenic fusion kinase is expressed in cells. However, it may be difficult to develop lineage tracing models to define the usual cell type of origin as FLC develops without chronic liver injury.

## Cell of origin of cHCC-ICC

Human cHCC-ICC is a rare and aggressive form of primary liver cancer that displays morphological features of both HCC and cholangiocarcinoma and is considered possibly HPC-derived.^89^^,^^132^ The HPC origin is favoured by gene expression studies demonstrating stem/progenitor features, downregulation of an HNF4α-driven hepatocyte differentiation program, and upregulation of genes associated with biliary commitment in a series of cHCC-ICC with stem cell features. Notably, TGFβ and Wnt/β-catenin were the main signalling pathways activated in the examined tumours.^133^ A comprehensive molecular characterisation of cHCC-ICCs, encompassing the whole histological spectrum of the disease, found that the cholangiolocellular carcinoma subtype of cHCC-ICC with stem cell features is defined by solely biliary features with no genomic characteristics of HCC, suggesting a biliary cell of origin for this entity.^134^ Importantly, in the 2019 World Health Organization histological classification system, the subtype of cHCC-ICC with stem cell features is no longer used.^135^ While the HPC origin seems to be a plausible explanation for the biphenotypic appearance, lineage tracing in animal models implies that these cells may come from HPCs, hepatocytes, or hepatoblasts.^90^^,^^91^^,^^114^

## Conclusions

Results in mouse models indicate that hepatocytes are likely the main source of cells replenishing the liver after injuries. Any hepatocyte may have the capacity to repopulate, but a preference for zone 2 hepatocytes, which may be the predominant cell in the liver anyway, exists to repopulate the liver during injury and homeostasis. However, BECs and HPCs can give rise to hepatocytes in certain types of severe, chronic liver injury. Further defining the molecular signalling involved in hepatocyte and HPC replenishment of liver parenchyma may provide strategies to improve healing after injuries, which in turn may prevent cancer formation.

Several studies also support hepatocytes as the predominant cell of origin of HCC. However, HPCs and foetal progenitor cells have been found to form HCC if they are induced to express oncogenic driver genes or in certain injury contexts.

Hepatocytes, cholangiocytes, and HPCs exhibit a high degree of plasticity and heterogeneity.[136], [137], [138], [139] Therefore, an important subject of future study is to address whether these epithelial cells can be directly transformed into cancer cells, or whether an intermediate progenitor or dedifferentiated state is required for tumorigenesis. Extrapolating from the data on the cell of origin for liver regenerative responses to injury, it is conceivable that prolonged injuries can induce conversion of HPCs to hepatocytes, which in turn form HCC. It may be a matter of duration of injury, the type of injury, and the types of oncogenic drivers that dictates which cells convert to HCC.

Although it is becoming increasingly clear that hepatocytes are the major cell of origin for HCC in animal models, further research is needed to clarify how specific subsets or ploidy states of hepatocytes, or type and length of liver injury, tend to contribute to tumourigenesis or tumour phenotype. Many of the genetic lineage tracing models studied in liver repopulation have not yet been tested for their contribution to hepatocarcinogenesis.

While lineage tracing in mice has clearly defined the cells of origin for regeneration and HCC under specific circumstances, it is difficult to extrapolate directly to the situation in humans. As it is impossible to perform Cre recombinase-based lineage tracing in the context of human disease, alternative methods should be explored, such as tracing based on DNA methylation status or mitochondrial DNA mutations elaborated using single cell sequencing.^140^^,^^141^

The major implication of better defining the cell of origin is to determine which cells to target to promote healthy regenerative responses to liver injuries, on the one hand, and, on the other, which cells to focus on for the primary or secondary prevention of HCC.

## Financial support

Related work in the authors’ laboratories was supported by the 10.13039/100000002National Institutes of Health (R03DK123543 to K.J.W. and R37CA225807 to S.S.) and by the 10.13039/100000997Arnold and Mabel Beckman Foundation (Beckman Young Investigator Award to K.J.W.).

## Authors’ contributions

AH, KJW, SS: conceptualization, writing – original draft, writing – review & editing.

## Conflicts of interest

The authors declare no conflicts of interest that pertain to this work.

Please refer to the accompanying ICMJE disclosure forms for further details.
